# Gut microbiota alterations in response to sleep length among African-origin adults

**DOI:** 10.1371/journal.pone.0255323

**Published:** 2021-09-08

**Authors:** Na Fei, Candice Choo-Kang, Sirimon Reutrakul, Stephanie J. Crowley, Dale Rae, Kweku Bedu-Addo, Jacob Plange-Rhule, Terrence E. Forrester, Estelle V. Lambert, Pascal Bovet, Walter Riesen, Wolfgang Korte, Amy Luke, Brian T. Layden, Jack A. Gilbert, Lara R. Dugas

**Affiliations:** 1 Microbiome Center, Department of Surgery, University of Chicago, Chicago, IL, United States of America; 2 Public Health Sciences, Parkinson School of Health Sciences and Public Health, Loyola University Chicago, Maywood, IL, United States of America; 3 Department of Psychiatry & Behavioral Sciences, Biological Rhythms Research Laboratory, Rush University Medical Center, Chicago, IL, United States of America; 4 Research Unit for Exercise Science and Sports Medicine, University of Cape Town, Cape Town, South Africa; 5 Solutions for Developing Countries, University of the West Indies, Mona, Kingston, Jamaica; 6 Department of Physiology, School of Medical Sciences, Kwame Nkrumah University of Science and Technology, Kumasi, Ghana; 7 University Center for Primary Care and Public Health (Unisanté), Lausanne, Switzerland; 8 Ministry of Health, Victoria, Republic of Seychelles; 9 Center for Laboratory Medicine, Canton Hospital, St. Gallen, Switzerland; 10 Jesse Brown Veterans Affairs Medical Center, Chicago, Illinois, United States of America; 11 University of California San Diego, San Diego, California, United States of America; 12 Division of Epidemiology & Biostatistics, School of Public Health & Family Medicine, University of Cape Town, Cape Town, South Africa; University of Naples Federico II, ITALY

## Abstract

Sleep disorders are increasingly being characterized in modern society as contributing to a host of serious medical problems, including obesity and metabolic syndrome. Changes to the microbial community in the human gut have been reportedly associated with many of these cardiometabolic outcomes. In this study, we investigated the impact of sleep length on the gut microbiota in a large cohort of 655 participants of African descent, aged 25–45, from Ghana, South Africa (SA), Jamaica, and the United States (US). The sleep duration was self-reported via a questionnaire. Participants were classified into 3 sleep groups: short (<7hrs), normal (7-<9hrs), and long (≥9hrs). Forty-seven percent of US participants were classified as short sleepers and 88% of SA participants as long sleepers. Gut microbial composition analysis (16S rRNA gene sequencing) revealed that bacterial alpha diversity negatively correlated with sleep length (p<0.05). Furthermore, sleep length significantly contributed to the inter-individual beta diversity dissimilarity in gut microbial composition (p<0.01). Participants with both short and long-sleep durations exhibited significantly higher abundances of several taxonomic features, compared to normal sleep duration participants. The predicted relative proportion of two genes involved in the butyrate synthesis via lysine pathway were enriched in short sleep duration participants. Finally, co-occurrence relationships revealed by network analysis showed unique interactions among the short, normal and long duration sleepers. These results suggest that sleep length in humans may alter gut microbiota by driving population shifts of the whole microbiota and also specific changes in Exact Sequence Variants abundance, which may have implications for chronic inflammation associated diseases. The current findings suggest a possible relationship between disrupted sleep patterns and the composition of the gut microbiota. Prospective investigations in larger and more prolonged sleep researches and causally experimental studies are needed to confirm these findings, investigate the underlying mechanism and determine whether improving microbial homeostasis may buffer against sleep-related health decline in humans.

## Background

Disrupted sleep has been associated with disturbances of hormone secretion and metabolism [1], as well as affecting physical, mental and emotional functions [2–7]. Recent data has shown that modern society as whole is severely sleep deprived, and that this chronic state of deprivation has consequences on the persons and society [8–11]. The underlying mechanisms of by which disrupted sleep patterns alter disease risk, especially cardiometabolic risks, have been demonstrated in several well-conducted sleep restriction experiments in healthy volunteers [12–15]. These include increased inflammatory markers [14], increased sympathetic nervous system activity [13], abnormal cortisol rhythmicity [13], alterations in appetite regulating hormones and food intake [16], and adipocyte dysfunction [15] which altogether contribute to insulin resistance, diabetes and obesity risks. Many of these findings have been confirmed in laboratory studies, demonstrating that disrupted sleep may be a risk factor for incident diabetes and obesity [17, 18]. According to the American Academy of Sleep Medicine and Sleep Research Society, the recommended sleep duration in adults is between 7–9 hours [19]. Despite strong data suggesting a causal association between disrupted sleep patterns and cardiometabolic risk, only a few sleep extensional studies have been conducted to date [20–23]. While preliminary evidence supports a possible benefit in glucose metabolism and dietary patterns, the ability to increase sleep time varied among participants [21–23]. Thus, development of preventive strategies based on new, modifiable risk factors is therefore imperative.

One such novel factor is the gut microbiota, important for regulating human physiology [24]. Structural and functional configurations of the gut bacterial community are associated with a series of metabolic and immune diseases, which are also adverse health consequences of disrupted sleep patterns [2, 25–28]. Emerging data from animal and human experimental studies have shown that sleep restriction or fragmentation led to significant changes in the structure of their intestinal microbial communities [29–31], although this was not consistently observed in all the studies related to sleep restriction and intestinal microbiota [32]. Structural changes in the gut microbiota may promote an increase in dietary fat intake and an increase in fat storage through a series of signal transductions [33]. Gut microbiota dysbiosis has recently been associated with systemic inflammation by producing butyrate with amino-acids (lysine, glutarate and 4-aminobytyrate/succinate) as substrates via the 4-aminobutyrate pathway, lipopolysaccharide (LPS) or hydrogen sulfide (H_2_S) [34–37], etc. Thus, alterations in gut microbiota may also contribute to the systemic inflammation which is a known consequence of disrupted sleep patterns [38].

Given the intersection between the gut microbiota, cardiometabolic disease and disrupted sleep patterns, it is possible that the gut microbiota is a potential mediator linking disrupted sleep and adverse health outcomes. Indeed, these associations between disrupted sleep patterns and changes in the gut microbiota community are not well understood. Notably, sleep-derived variations in the composition of gut microbiota has only been explored in small groups involving experimental sleep restriction [31]. Studies in large cohorts that explore this association across different geographic regions, with their own lifestyle and geographic idiosyncrasies could help elucidate the general characteristics that determine the differences in the microbiota across gradients of sleep duration. Therefore, to examine the potential impact of sleep disruption on the gut microbiota, this study leveraged African-origin participants enrolled in the Modeling the Epidemiologic Transition Study” (METS) cohort to investigate the structural and predicted metabolic dynamics of the gut (stool-derived) microbiota as a function of sleep quality in 655 adults of African descent aged 22 to 45 years.

## Results

### Participant characteristics

The first METS cohort consisted of 2,506 participants enrolled in December 2010 and January 2011, and 655 participants from this original cohort were additionally asked to provide stool samples for this current microbiome study. These 655 participants were recruited from Ghana (N = 196), South Africa (N = 176), Jamaica (N = 92) and US (N = 191) (Table 1). Prevalence of overweight and obesity was significantly higher in the US cohort (81.3%) when compared to the other sites (i.e., Ghana, 33.2%, South Africa, 55.7%, Jamaica 65.2%). South Africans and Americans had significantly higher blood pressure than Ghanaians and Jamaicans (Table 1). Americans also slept the least number of hours (6.7 ± 1.4 hours) compared to Ghanaians (7.9 ± 1.4 hours, p<0.001), Jamaicans (7.3 ± 2.1 hours, p<0.05), and South Africans (10.5 ± 1.7 hours, p<0.001). The normal range of sleep for adults defined by the American Academy of Sleep Medicine is 7–9 hours [19], therefore if a participant slept 7–9 hours, then he/she was identified as a “normal sleeper”. If a participant slept<7 hours of per 24-hour period, then he/she was considered to be a “short sleeper” and if >9 hours: a “long sleeper”. Overall, the US had the greatest proportion of short sleepers, while South Africa had highest proportion of long-sleepers. Table 2 presents a summary of the distribution across the 3 groups.

**Table 1:** Participant characteristics and CM risk factors by site (mean, std. dev).

	Ghana			South Africa			Jamaica			United States			Overall		
	(N = 196)			(N = 175)			(N = 90)			(N = 191)			(N = 652)		
Sleep Category	Short	Normal	Long	Short	Normal	Long	Short	Normal	Long	Short	Normal	Long	Short	Normal	Long
N	N = 28	N = 113	N = 55	N = 3	N = 18	N = 154	N = 33	N = 33	N = 24	N = 90	N = 86	N = 15	N = 154	N = 250	N = 248
Age (y)	37.4 ± 6.1	36.0 ± 6.4	34.6 ± 7.2	37.7 ± 3.1	36.6 ± 5.1	**32.9 ± 6.0** **	32.3 ± 6.2	34.3 ± 6.4	35.8 ± 5.6	36.2 ± 5.9	35.8± 6.7	34.3 ± 6.5	35.6 ± 6.2	35.7 ± 6.4	**33.6 ± 6.3** **
Weight (kg)	62.7 ± 7.7	63.7 ± 12.8	62.3 ± 12.3	84.4 ± 21.7	87.5 ± 23.6	**74.8 ± 19.9** *	81.3 ± 21.3	83.6 ± 23.8	74.5 ± 16.0	95.5 ± 27.4	94.1 ± 22.8	86.9 ± 22.2	86.3 ± 26.6	78.5 ± 23.5	**72.7 ± 19.2** *
Height (cm)	163.4 ± 8.7	161.4 ± 7.2	161.3 ± 7.9	168.4 ± 8.1	166.2 ± 8.3	163.3 ± 7.6	172.2 ± 10.0	166.1 ± 8.3	161.1 ± 12.2	170.0 ± 9.5	169.3 ± 7.2	169.2 ± 8.0	169.2 ± 9.8	165.1 ± 8.2	163.0 ± 8.4
BMI (kg/m^2^)	23.5 ± 2.9	24.6 ± 5.6	24.0 ± 4.6	30.3 ± 10.5	32.1 ± 9.6	**28.3 ± 8.0** *	27.5 ± 7.2	30.5 ± 9.3	29.6 ± 10.5	33.2 ± 9.6	32.9 ± 8.1	30.5 ± 8.1	30.2 ± 9.1	28.8 ± 8.3	**27.6 ± 7.9** *
Sleep hours (hrs/night)	5.6 ± 0.69	7.6 ± 0.48	9.5 ± 0.77	5.7 ± 0.58	7.7 ± 0.46	11.0 ± 1.3	5.1 ± 1.0	7.6 ± 0.50	9.9 ± 1.1	5.4 ± 0.72	7.5 ± 0.51	9.2 ± 0.41	5.4 ± 0.80	7.6 ± 0.50	10.4 ± 1.4
SBP (mm Hg)	114.0 ± 9.9	113.6 ± 14.9	111.0 ±12.0	129.2 ± 22.5	118.0 ± 13.8	125.1 ± 21.0	112.1 ± 10.5	113.6 ± 15.0	112.1 ± 12.1	123.1 ± 18.9	123.5 ± 15.8	122.9 ± 20.0	119.2 ±16.8	117.3 ± 15.8	120.6 ± 19.6
DBP (mm Hg)	65.3 ± 7.4	67.1 ± 12.1	66.2 ± 10.3	81.6 ± 20.0	75.0 ± 11.3	79.9 ± 12.9	66.3 ± 8.6	80.4 ± 55.2	68.8 ± 8.1	79.9 ± 14.3	81.2 ± 11.8	80.0 ± 13.9	74.4 ± 14.0	74.3 ± 23.6	75.8 ± 13.4
HDL (mg/dL)	42.3 ± 14.5	46.4 ± 11.4	47.5 ± 16.8	40.0 ± 11.8	49.8 ± 15.3	50.1 ± 15.0	**-**	**-**	**-**	52.5 ± 15.7	51.8 ± 14.9	52.3 ± 13.1	49.7 ± 16.0	48.8 ± 13.4	49.6 ± 15.3
LDL (mg/dL)	94.4 ± 26.7	102.2 ± 29.6	101.1 ± 29.4	83.7 ±37.7	93.5 ± 35.0	93.0 ± 32.4	**-**	**-**	**-**	107.6 ± 30.9	114.4 ± 40.0	116.7 ± 34.1	103.8 ± 30.5	105.1 ± 34.8	**96.6 ± 32.3** *
Trigs (mg/dL)	77.2 ± 33.7	82.3 ± 33.4	84.2 ± 43.5	**135.8 ± 53.4** *	76.3 ± 38.2	88.7 ± 56.5	**-**	**-**	**-**	91.2 ± 50.9	103.0 ± 73.6	115.1 ± 46.6	89.0 ± 48.0	90.0 ± 54.1	89.4 ± 53.3

If data were normal, student t- testing was used to analyze differences between short and long sleepers with normal sleepers (i.e. short sleepers were compared to normal sleepers and long sleepers were compared to normal sleepers) within each site and overall for each characteristic. Non-parametric testing was used for non-normal data.

*p<0.05

**p<0.01.

**Table 2:** Participant characteristics by site (N,%).

	Ghana			South Africa			Jamaica			United States			Overall		
	(N = 196)			(N = 175)			(N = 90)			(N = 191)			(N = 652)		
Sleep Category	Short	Normal	Long	Short	Normal	Long	Short	Normal	Long	Short	Normal	Long	Short	Normal	Long
N	N = 28	N = 113	N = 55	N = 3	N = 18	N = 154	N = 33	N = 33	N = 24	N = 90	N = 86	N = 15	N = 154	N = 250	N = 248
**Sex** ^**2**^															
Women	15, 53.6%	74, 65.5%	38, 69.1%	1, 33.3%	12, 66.6%	98, 63.6%	15, 45.5%	27, 81.8%	19, 79.2%	44, 48.9%	50, 58.1%	8, 53.3%	75, 48.7%	163, 64.9	163, 65.7%
Men	13, 46.4%	39, 34.5%	17, 30.9%	2, 66.7%	6, 33.3%	56, 36.4%	18, 54.6%	6, 18.2%	5, 20.8%	46, 51.1%	36, 41.9%	7, 46.7%	79, 51.3%	88, 35.1	85, 34.3%
**BMI class**															
^1^Normal weight	21, 75.0%	74, 65.5%	36, 65.5%	1, 33.3%	6, 33.3%	71, 46.1%	13, 39.4%	9, 27.3%	9, 37.5%	20, 22.2%	12, 14.0%	4, 26.7%	55, 35.7%	101, 40.2%	120, 48.4%
^1^Overweight	6, 21.4%	24, 21.2%	11, 20.0%	1, 33.3%	2, 11.1%	24, 15.6%	11, 33.3%	10, 30.3%	6, 25.0%	23, 25.6%	24, 27.9%	5, 33.3%	41, 26.6%	61, 24.3%	46, 18.6%
^1^Obese	1, 3.6%	15, 13.3%	8, 14.6%	1, 33.3%	10, 55.6%	59, 38.3%	9, 27.3%	14, 42.4%	9, 37.5%	47, 52.2%	50, 58.1%	6, 40.0%	58, 37.7%	89, 35.5%	82, 33.1%
Smokers	0, 0%	1, 0.9%	3, 5.5%	0, 0%	5, 26.3%	43, 27.9%	5, 15.2%	4, 12.1%	1, 4.2%	32, 35.6	31, 36.1	9, 60.0	31, 27.0%	37, 17.4%	55, 24.7%

*χ*^2^ testing used to analyze differences between normal and short sleepers within each site and overall for each characteristic.

^**1**^Where “normal weight”, “overweight” and “obese” are defined as BMI<25 kg/m^2^, BMI≥25 kg/m^2^-<30 kg/m^2^ and BMI≥30 kg/m^2^, respectively.

^**2**^*χ*^2^ testing reached significance in Jamaica (p<0.05) and “Overall” (p<0.001).

### Sleep length significantly impact gut-derived bacterial diversity and composition

**Fig 1:**
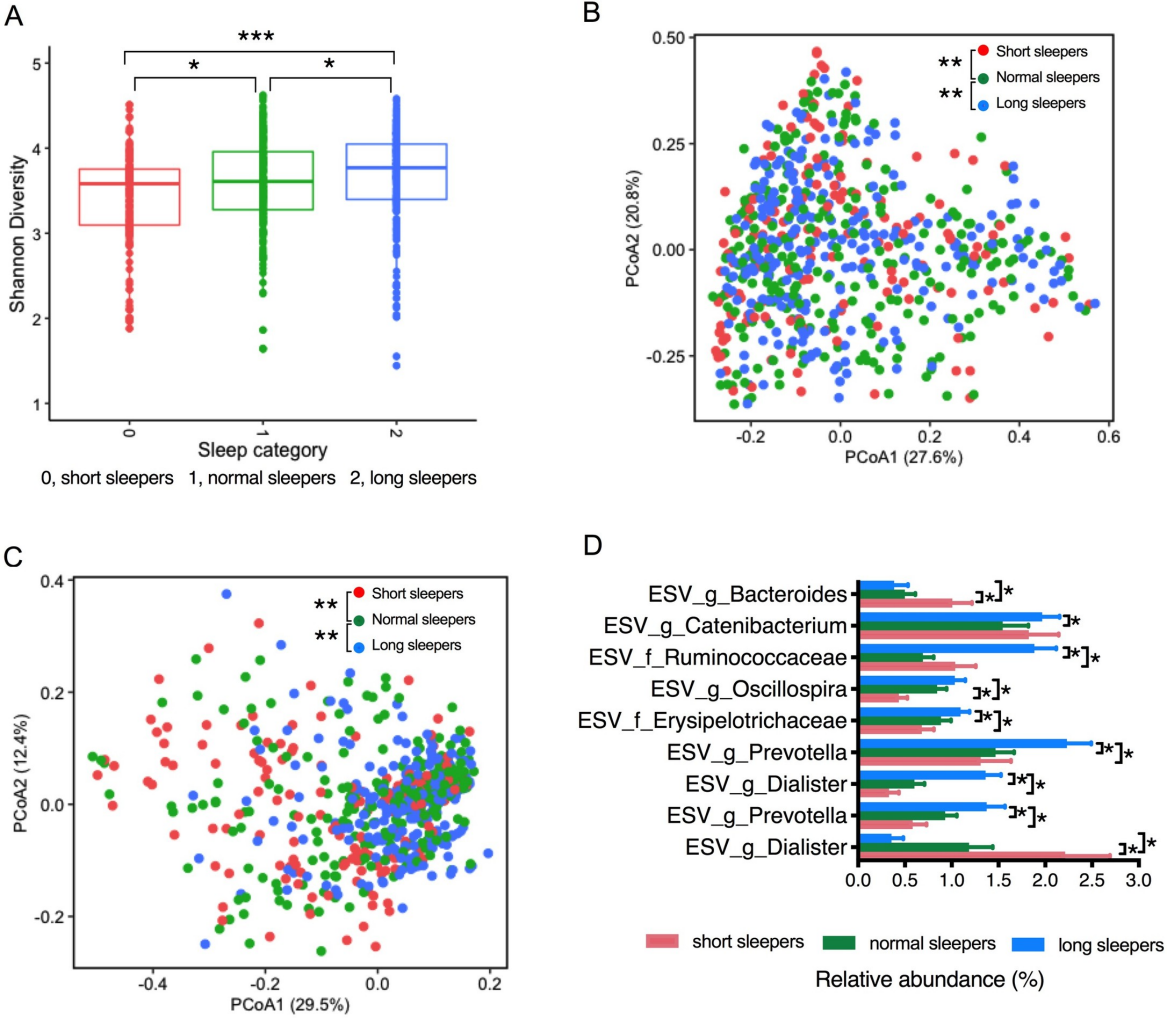
Sleep length can significantly impact intestinal microbiota community structure. (A) Alpha diversity (Shannon index) between different sleep groups; * p < 0.05; *** p < 0.001. (B) Beta diversity analysis (weighted UniFrac distance) between different sleep groups; (C) Beta diversity analysis (unweighted UniFrac distance); and (D) Differential ESV abundance among short, normal and long sleepers, adjusted for BMI, age, gender and countries. ESVs with relative abundance ≥ 1% in at least one group shown. Data shown are means ± S.E.M.; * p(fdr-corrected) < 0.05. fdr, false discovery rate.

We used 16S rRNA amplicon sequencing to define shifts in microbial community structure and composition associated with sleep length. A total of 10,805,592 16S ribosomal RNA gene sequences with good quality were generated from the 655 fecal samples, which clustered into 6, 909 Exact Sequence Variants (ESVs). Alpha diversity was calculated by Shannon’s diversity index diversity [39], which is a quantitative measure of community richness, and observed otu diversity, which a qualitative measure of community richness [39]. Gut microbial alpha diversity was significantly different between normal sleepers, short sleepers, and long sleepers, whereby short sleepers had a significantly lower alpha diversity, and long sleepers had a significantly higher alpha diversity compared with normal sleepers (Fig 1A, Shannon index, p<0.05. S1 Fig. Observed otu diversity). Principal coordinate analysis (PCoA) was performed based on weighted and unweighted UniFrac distances, a method for computing differences between microbial communities based on phylogenetic information [40]. 1000 reads/samples were considered to calculate the inter-individual beta diversity dissimilarity. Weighted UniFranc considered both ESVs presence/absence and abundance distances, and unweighted UniFrac only considered ESVs presence/absence. Permutational multivariate analysis of variance (PERMANOVA, R function adonis (vegan, 999 permutations)) was used to analyze statistical differences in beta diversity [41]. Similarly, we found that sleep length significantly contributed to the inter-individual beta diversity dissimilarity in Exact Sequence Variants (ESV)-level gut microbial composition, both in the weighted and unweighted UniFrac dissimilarity index (permutational multivariate analysis of variance (PERMANOVA), p<0.001) (Fig 1B and 1C). Pairwise comparation based on permutation tests showed that both weighted and unweighted UniFrac distances between samples within short sleepers and long sleepers are significantly different from that between samples within normal sleepers (p<0.01). These data suggest that ESV features contribute markedly to the dissimilarity in microbial community structure in response to sleep length. However, neither alpha diversity (S2 Fig) nor beta diversity (weighted and unweighted UniFrac dissimilarity index, S3 and S4 Figs) within each country site (Ghana, South Africa, Jamaica or US) were significantly different among participants with different sleep length (p > 0.05), which is most likely due to small size of the cohorts. Since geographic location contributed significantly to the distribution of CM risk factors and sleep duration, e.g. Americans have the highest prevalence of overweight and obesity and slept the least number of hours, when compared to the other sites, we also investigate its effect in gut microbial community structure. Gut microbial alpha diversity was significantly lower in Americans compared to Ghanaians, Jamaicans, and South Africans (p<0.001) (S5A Fig). Geographic location also significantly contributed to the inter-individual beta diversity dissimilarity gut microbial composition in the weighted UniFrac dissimilarity index (PERMANOVA, p<0.001) (S5B Fig). These observations suggest that a country-related physiological or environmental influence change in the host is conducive to a less complex microbiota.

The taxonomic features that differentiated the gut microbiota among short, normal and long sleepers were determined using Analysis of Composition of Microbiota (ANCOM, adjusted for country, age, gender and BMI, p (false discovery rate (fdr)-corrected) < 0.05) (Fig 1D). An ESV assigned to genus *Dialister* was significantly enriched in short sleepers, while another ESV also assigned to genus *Dialister* was at a significant greater proportion in the long sleepers, compared to the normal sleepers. 2 ESVs belonging to genus *Prevotella* were both more abundant in the long sleepers, compared to short and normal sleepers. Furthermore, long sleepers were also significantly enriched with 4 ESVs belonging to family Erysipelotrichaceae, family Ruminococcaceae, genus *Oscillospira*, and genus *Catenicacterium*, compared to the other two groups. And short sleepers had significantly higher proportion of one ESV from genus *Bacteroides*, compared to normal and long sleepers (Fig 1D).

Piphillin [42] was used to predict the gene abundances for gut microbial metabolic pathways based on the 16S rRNA gene abundance. Specifically, we wanted to determine the differences in the predicted abundance of gut microbial genes associated with butyrate metabolism (via less-dominant pathway-the 4-aminobutyrate pathway, include amino-acids (lysine, glutarate and 4-aminobytyrate/succinate) as substrates [38], H_2_S and LPS synthesis between short, normal and long sleepers, which are involved in the low-grade systemic inflammation in metabolic diseases [34–37]. Predicted genes involved in butyrate biosynthesis pathways showed that only 3,5-diaminohexanoate dehydrogenase (K18012) and 3-keto-5-aminohexanoate cleavage enzyme (K18013) in the Lysine pathway was enriched in short sleepers, compared to normal sleepers and long sleepers (S6 Fig; p_fdr_<0.05, adjusted for age, gender, country and BMI). No statistically significant differences in the genes involved in the LPS or H_2_S synthesis pathway were observed between short sleepers and normal sleepers (p_fdr_>0.05, adjusted for age, gender, country and BMI).

**Fig 2:**
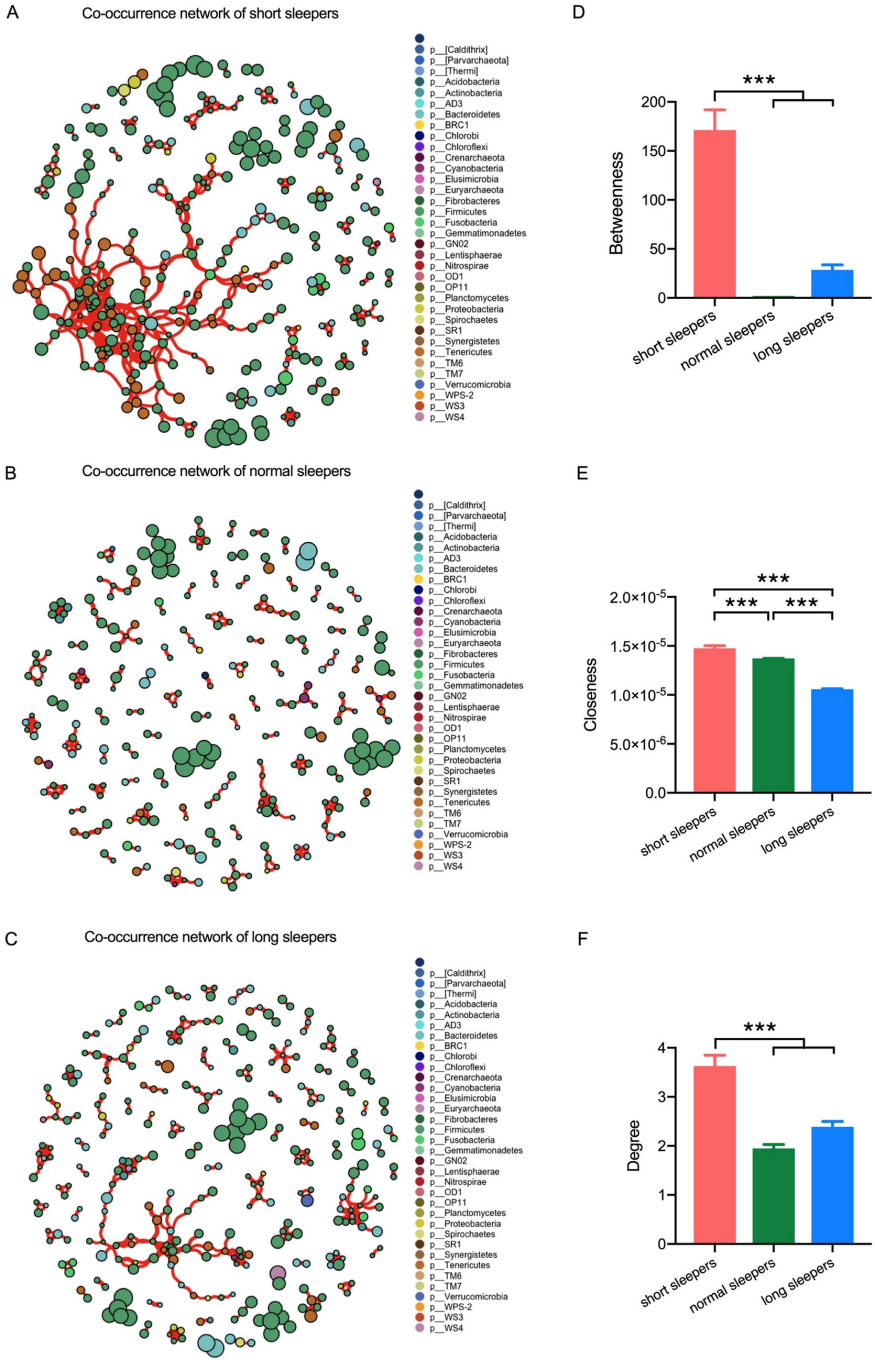
The network analysis revealing the co-occurrence patterns among short sleepers, normal sleepers and long sleepers. A connection represents a strong (Spearman’s correlation coefficient *ρ*>0.6) and significant (*P* <0.05) correlation. A-C, co-occurrence network of short sleepers (A) normal sleepers (B) and long sleepers (C) The nodes represented unique ESV feature in the data sets. The size of each node is proportional to the relative abundance. Node color corresponds to phylum taxonomic classification. The edge thickness is equivalent to the correlation values. D-F, Topological features for each node in the network, (D) betweenness centralization, (E) closeness centrality and (F) node degree values. *** p< 0.0001.

A correlation network was constructed by calculating Spearman correlations between ESVs using the gut microbiota of short, normal and long sleepers’ datasets, and only robust correlations were considered for network construction (-0.60 ≥ ρ≥ 0.60, p_fdr_ <0.05). The vertices in this network represent ESVs and the edges that connect these nodes represent correlations between ESVs. The network of the short and long sleepers’ microbiota has a higher number of vertices and edges (short sleepers, 312 vertices and 566 edges, long sleepers, 315 vertices and 376 edges) in comparison to that of normal sleepers (272 vertices and 265 edges) (Fig 2A–2C). Using the co-occurrence network outlined above, we also examined whether ESVs associated with a special sleep length exhibited unique node-level topological features. This feature set included betweenness centrality (a measure of centrality based on the number of times a node acts as a bridge along the shortest path between two other nodes.), closeness centrality (a measure of centrality based on the sum of the length of the shortest paths between this node and connected nodes), and degree (the number of connections a single node has). The betweenness centrality feature was used to measure the centrality of each node in the network. Significantly higher betweenness centrality scores were observed for ESVs associated with short sleepers’ microbiota than those associated with normal or long sleepers’ microbiota (p_fdr_ <0.05, Fig 2D). This suggests that the gut microbes from the short sleepers were more often located in central positions within the network, compared to those from the normal sleepers. We next examined additional node-level topological measures, degree and closeness, for each ESV in the co-occurrence network. Degree and Closeness only take into account only the immediate neighborhood of ESVs, and hence capturing a different aspect of network topological features. Closeness and degree of ESVs associated with short sleepers’ microbiota were also significantly higher than those associated with normal or long sleepers’ microbiota (p <0.001, Fig 2E and 2F). Thus, co-occurrence relationships revealed by network analysis showed unique interactions among the short, normal and long duration sleepers, which the gut microbiota of short sleepers had a closer connected network, compared to those of normal and longer sleepers.

## Discussion

Recently, there has been some evidence that a relationship between disrupted sleep and the gut microbiota exists because both sleep alterations as well as a gut dysbiosis are associated with similar diseases [24, 43]. However, this relationship is not well understood, and studies have been limited by small sample sizes. Here, we performed the first investigation into how sleep length may alter the human gut microbiota in a large cohort of African-origin adults. Overall, we demonstrate that the structure of the gut microbiota is remarkably impacted by sleep length. Indeed, it may well be that many of the adverse effects that have been observed in sleep disorder may be due in part to gut microbial dysbiosis.

Most of the health decline in humans have been reported to be associated with reduced overall bacterial community diversity and richness [44–47], which is identified as one of the characters of gut microbial dysbiosis. In our current study, we saw that sleep length significantly associated with the alpha diversity in humans. Participants with short sleep duration exhibited lower alpha diversity; however, Participants with long sleep duration showed higher alpha diversity compared to those with normal sleep duration, which implied that not only too low, but too high gut bacterial community diversity may also induce the gut microbial dysbiosis. This suggest that chronic sleep disruption may also promote an intestinal microbial dysbiosis [29–31]. Certainly disrupted sleep may serve as another novel environmental factor that induce gut bacterial diversity, besides diet, urbanization, and other lifestyle changes in modern society [48].

In this study, we observed an increased relative abundance of one ESV assigned to genus *Dialister* in the gut community of participants with short or long sleep duration separately, compared to normal length sleepers. An increase of the species or strains in this genus, has been found to be associated with many kinds of diseases in humans, such as spondyloarthritis [49], periodontitis [50], inflammatory bowel disease and their unaffected relatives [51, 52], pulmonary disease [53] and so on. Such changes may promote increased chronic inflammatory state and could therefore constitute a possible mechanism through which chronic sleep loss can increase the risk of human diseases. We also observed decreased levels of two ESVs assigned to the genus *Prevotella* in the gut community of participants with short sleep duration. Reduced incidence of genus *Prevotella* have been associated previously with autistic children [54], and Parkinson’s disease [55]. However, one ESV assigned to genus *Prevotella* was significantly higher in the gut community of participants with long sleep duration, and it should also be noted that there are a handful of studies that suggest that *Prevotella* are significantly enriched in patients with metabolic diseases [56–58]. This was the first study to show that short sleep participants with harbored lower proportions of ESVs assigned to genus *Prevotella*, while long sleepers harbored higher proportions of ESVs assigned to genus *Prevotella*. Furthermore, this study also showed that participants with long sleep duration were also significantly enriched with 4 ESVs belonging to family Erysipelotrichaceae, family Ruminococcaceae, genus *Oscillospira*, and genus *Catenicacterium*, compared to the participants with normal or short sleep duration. The importance of Erysipelotrichaceae in inflammation-related disorders of the gastrointestinal tract is highlighted by the fact that they have been found to be enriched in individuals with colorectal cancer or IBD in both human and mouse studies [59–62]. In addition, higher levels of Erysipelotrichaceae have been observed in human disease related to metabolic disorders [63–65]. However, several recent studies have observed that members of the *Oscillospira* genus were highly enriched in lean subjects, and positively associated with the leanness in human and mouse studies [66, 67], and decreased in individuals with inflammatory diseases, nonalcoholic steatohepatitis [68], Crohn’s disease [69]. Members of the family Ruminococcaceae, which are involved in degrading dietary indigestible fibers and polysaccharides and in producing butyrate, were also important in improving weaning weight [70]. An increase of the relative abundance in the genus *Catenicacterium* has been found to be associated with obesity with metabolic syndrome [71], however, it was also shown that cholorectal cancer patients harbor less *Catenicacterium* in the gut [72]. We also observed that participants with short sleep duration had significantly higher proportion of one ESV from genus *Bacteroides*, compared to normal and long sleepers. *Bacteroides*, an abundant genus in the intestines of mammals, play essential roles in host immunity, glucose and lipid metabolism and the prevention or induction of diseases [73–80]. The specific role the members in genus *Bacteroides* found in this study requires more experimental evidence to interpret their role. Given that many members of these gut microbiota above have not yet been grown in culture and more experimental evidence need to be collected, it remains a conundrum in gut microbiota research and the association between gut microbiota and sleep disruption. However, in light of the strong evidence linking these gut microbiol members to human health and disease, these bacteria should be cultivated to characterize both genome and function to investigate its potential link to the sleep disruption in future studies.

Similarly, we observed that sleep length was associated with the gut microbiota functionality. The predicted functional potential of the bacterial communities showed significant elevated abundance of two predicted enzymes involved in the butyrate synthesis via lysine pathway in short sleep participants. It has shown the beneficial effects of butyrate on host homeostasis by enhancing gut barrier function and reducing inflammation [81], however, butyrate synthesis via less-dominant pathway-the 4-aminobutyrate pathway, include amino-acids (lysine, glutarate and 4-aminobytyrate/succinate) as substrates, can produce pro-inflammatory byproducts and increase the risk of human diseases [37].

The co-occurrence networks generated for the gut microbiota from participants with short, normal and long sleep duration showed significant differences in the node-level features, specifically between the participants with short sleep duration, compared to the other 2 cohorts. Participants with short sleep duration have more being center nodes, that were closely connected within the network, and nodes with increasing influence in connecting different parts of the network, compared to the less connected and centered co-occurrence networks, as found in participants with normal and long sleep duration. Co-occurrence relationships revealed by network analysis showed unique interactions among the participants with different duration.

At present, it is still not known that if there’s a causal relationship between disrupted sleep and disrupted gut microbiota in humans. It is not clear there is a common mechanism mediates the effects of disrupted gut microbiota and sleep disorder either. There are several potential mechanisms that are listed in previous publications and reviews in the field, such as alterations in interaction of gut microbiome with bile acid metabolism [82, 83], microbiota-mediated molecular signaling, like SCFA [84–86] or altered nutrient uptake through the intestinal wall [87, 88], etc. Chronic inflammation also appears to be an important mechanism for the deleterious health consequences of insufficient sleep and gut microbial dysbiosis, as exemplified by increased risk and severity of obesity and related other metabolic syndrome [89], cardiovascular disease [90], and inflammatory bowel disease [91]. Notably, when considered alongside previous studies, the current research suggests that disrupted sleep may be associated with dysbiosis of gut microbiota in humans (including overall decreased diversity, changed abundance of some taxa and unique co-occurrence network). However, this study only observes an association and doesn’t prove causality between the sleep disruption and gut microbial dysbiosis with the composition of gut microbiota. In future, prospective investigations in larger and more prolonged sleep researches and causally experimental studies are needed to confirm these findings, investigate the underlying mechanism and determine whether improving microbial homeostasis may buffer against sleep-related health decline in humans. Lastly, considering that the significant geographically difference of sleep duration and the microbial composition showed in this study, the geographic effect need to be considered to investigate the potential relationship between sleep disruption and gut microbiota dysbiosis in the future studies

Our study is not without several limitations, including that sleep lengths were self-reported and do not include the time of day when sleep occurred. In fact, a single question was asked about sleep, thus excluding an evaluation of sleep. However, these weaknesses were balanced by several study strengths, including the large sample size, the use of cohorts from diverse countries and measured clinical data. Future directions of this study will include increasing the number of questions asked about sleep routines and quality. Also sleep will be objectively measured using sleep monitors.

## Conclusion

To our knowledge, our study is the first to provide information in a large human cohort that sleep length alters gut microbiota. The changes in the structural and functional dysbiosis of microbiota that we observed in the present study could be due, at least in part, to sleep length. These findings should be considered along with the limitations of the study. The sleep disorder-gut microbiota connection will require numerous larger studies, including longitudinal studies, to fully elucidate the role of the gut microbiota in relation to sleep length and associated deleterious health consequences. Similarly, further animal studies are needed to understand causal mechanisms by which sleep duration affects the gut microbial dysbiosis. Metagenomic sequencing might also need to be considered to determine whether disrupted sleep patterns functionally alter gut microbiota.

## Materials and methods

### Participant selection

The original METS cohort consisted of 2,506 male and female participants of African descent aged 25–45 years old. Participants were enrolled in METS between January 2010 and December 2011 and followed annually for three years. A detailed description of the METS protocol has previously been published [92]. For the current study of METS, the microbiome study, both fecal and saliva samples were collected in 2014 from 655 and 620 male and female participants from the first METS cohort, respectively, from Ghana (N = 196), South Africa (N = 176), Jamaica (N = 92) and the US (N = 191). These sites were chosen as they span the epidemiologic transition, and each site is at a different stage of development as defined by the United Nation’s Human Development Index. Exclusion criteria included self-reported infectious disease (e.g., HIV), pregnancy, current breast-feeding and inability to participate in normal physical activities. METS was approved by the Institutional Review Board of Loyola University Chicago, IL, US; the Committee on Human Research Publication and Ethics of Kwame Nkrumah University of Science and Technology, Kumasi, Ghana; the Research Ethics Committee of the University of Cape Town, South Africa; the Board for Ethics and Clinical Research of the University of Lausanne, Switzerland; and the Ethics Committee of the University of the West Indies, Kingston, Jamaica. All study procedures were explained to participants in their native languages, and participants provided written informed consent after being given the opportunity to ask any questions.

### Anthropometric and biochemical measurements and stool collection

All measurements were performed by site staff in their local research clinics. Participants wore light clothing and no shoes when weight and height were measured. Participants were asked to provide an early morning fecal sample, using a standard collection kit (EasySampler stool collection kit, Alpco, NH) at their home. Fecal samples were immediately brought to the site clinics and stored at -80°C. Participants were asked to fast 10–12 hours prior to clinic visit, so that fasting capillary glucose concentrations could be determined using finger stick (Accu-check Aviva, Roche).

### Sleep and demographic data

Sleep, age and smoking data were obtained via questionnaire. Participants were asked how many hours they slept each night. Neither the times at which they slept nor whether they slept during the day or evening was recorded.

### Statistical analysis

Statistical analyses for Tables 1and 2 were performed in Stata (version 12, Manufacturer, College Station, TX, USA). Table 1 summarized continuous participant characteristics and risk factors using mean ± SD, and Table 2 summarized dichotomous and categorical variables using proportions which were presented as *N*, %. Comparisons of participant characteristics and risk factors within sites and overall were performed using t-tests for normally distributed continuous variables, Wilcoxon rank-sum scores for non-normally distributed continuous variables and Pearson’s chi-squared test for categorical variables at a significance level (α) of 0.05.

### DNA isolation and 16S ribosomal RNA (rRNA) gene sequencing

The microbial genomic DNA from the human stool samples was extracted using the DNeasy PowerSoil DNA Isolation Kit (Qiagen) (Mo Bio Laboratories, Carlsbad, CA, USA) following the protocol of Flores et al [93]. The V4 region of 16S rRNA gene was amplified and sequenced using the Illumina MiSeq platform [94]. The primers used for amplification (515F-806R) contain adapters for MiSeq sequencing and single-end barcodes allowing pooling and direct sequencing of PCR products [95]. Each 25 μl PCR reaction contained the following mixture: 12 μl of MoBio PCR Water (Certified DNA-Free; Mo Bio Laboratories), 10 μl of 5-Prime HotMasterMix (1×), 1 μl of forward primer (5 μM concentration, 200 pM final), 1 μl of Golay Barcode Tagged Reverse Primer (5 μM concentration, 200 pM final), and 1 μl of template DNA [96]. The conditions for PCR were as follows: 94 °C for 3 min to denature the DNA, with 35 cycles at 94 °C for 45 s, 50 °C for 60 s, and 72 °C for 90 s, with a final extension of 10 min at 72 °C to ensure complete amplification. Amplicons were quantified using PicoGreen (Invitrogen, Grand Island, NY, USA) assays and a plate reader, followed by clean-up using UltraClean® PCR Clean-Up Kit (Mo Bio Laboratories) and then quantification using Qubit readings (Invitrogen). The 16S rRNA gene samples were sequenced on an Illumina MiSeq platform (2 × 150 paired-end sequencing, V3 chemistry) at Argonne National Laboratory core sequencing facility according to Earth Microbiome Project (EMP) standard protocols [97].

### 16S rRNA gene sequencing data preprocessing and analysis

Raw sequences were pre-processed, quality filtered and analyzed using the next-generation microbiome bioinformatics platform (QIIME2 version 2019.1 pipeline) according to the developer’s suggestion [98]. We used the DADA2 algorithm [99] a software package wrapped in QIIME2, to identify exact sequence variants (ESVs). Quality control, filtering low quality regions of the sequences, by truncating them to 120 base pair length, identification and removal of chimera sequences, merging paired end reads, which yielded the ESV feature table (ESV table). Chloroplast and mitochondrial DNA were removed. Alpha and beta-diversity analyses were performed in R using the *phyloseq* package [100]. Alpha diversity was calculated by Shannon’s diversity index diversity [39]. Results were adjusted for BMI, age, gender and country. Principal coordinate analysis (PCoA) was performed based on weighted and unweighted UniFrac distances, a method for computing differences between microbial communities based on phylogenetic information [40]. Weighted UniFranc considered both ESVs presence/absence and abundance distances, and unweighted UniFrac only considered ESVs presence/absence. Permutational multivariate analysis of variance (PERMANOVA, R function adonis (vegan, 999 permutations)) was used to analyze statistical differences in beta diversity [41]. Pairwise tests were performed to determine which specific pairs of groups (e.g., normal sleepers and short sleepers) differ from one another using the beta-group-significance command with the—p-pairwise parameter in QIIME2.

For taxonomic comparisons, relative abundances based on all obtained reads were used. We used the QIIME2 plugin “q2-feature-classifier” and the Naïve Bayes classifier that was trained on the metagenome annotation package Greengenes13.8 99% operational taxonomic units (OTUs) full-length sequences to obtain the taxonomy for each ESV [101]. Significantly differential ESVs were determined using the statistical framework called analysis of composition of microbiomes (ANCOM) [102] for two group comparisons. Benjamini–Hochberg false discovery rate (fdr) correction was used to correct for multiple hypothesis testing [103]. Results were adjusted for BMI, age, gender and country.

### Metagenome functional predictions of the microbial pathways

We used Piphillin algorithm to predict the functional profiles of the microbiome [42]. Briefly, this algorithm uses direct nearest-neighbor matching between 16S rRNA gene sequencing datasets and microbial genomic databases to infer the metagenomic content of the samples. Gene prediction was performed on ESVs table using online Piphillin (http://secondgenome.com/Piphillin.).), with KEGG (2017) as reference database and 97% identity cut-off. Predicted gene content and gene copy numbers within each genome were retrieved and classified in terms of KEGG orthology (KOs) [104]. Results were adjusted for BMI, age, gender and country. Statistical analyses were performed in R. Student’s t-test (normally distributed) or Mann-Whitney U test (not normally distributed) was used for to detect differentially abundant KOs between two groups. FDR correction was used to correct for multiple hypothesis testing.

### Network construction and topological feature analysis

To visualize the correlations in the network interface, we constructed a correlation matrix by calculating all possible pairwise Spearman’s rank correlations between the normal-, short- and long sleepers in the present study. To reduce rare ESVs in the data set, we removed ESVs with relative abundances less than 0.01% of the total number of sequences. A correlation between two ESVs was considered statistically robust if the Spearman’s correlation coefficient (*ρ*) was >0.6 and the *P*-value was <0.05. To reduce the chances of obtaining false-positive results, the *P*-values were adjusted with a multiple testing correction using the Benjamini–Hochberg method [103]. The robust pairwise correlations formed their co-occurrence networks. The nodes in this network represent ESVs and the edges that connect these nodes represent correlations between ESVs. Network analyses were performed in R environment using VEGAN [41], and igraph packages [105]. We calculated topological features for each node in the network with the igraph package [105]. This feature set included betweenness centrality (the number of shortest paths going through a node), closeness centrality (the number of steps required to access all other nodes from a given node), and degree (the number of adjacent edges). The betweenness centrality feature was used to measure the centrality of each node in the network.

## Supporting information

S1 Fig Alpha diversity (observed otu) from 16S rRNA gene sequence data between different sleep groups; ** p < 0.01; *** p < 0.001. (JPG)

S2 Fig Alpha diversity analysis (Shannon index) from 16S rRNA gene sequence data shows no difference among normal sleepers (1) and short sleepers (0) and long sleepers (2) in each country site. (**A**) USA, the United States of America, (**B**) RSA, South Africa (**C**) Ghanaian and (**D**) Jamaican populations.
(JPG)

S3 Fig Beta diversity analysis (weighted UniFrac distance metric) from 16S rRNA gene sequence data shows no difference among normal sleepers (1) and short sleepers (0) and long sleepers (2) in each country site. (**A**) USA, the United States of America, (**B**) RSA, South Africa (**C**) Ghanaian and (**D**) Jamaican populations.
(JPG)

S4 Fig Beta diversity analysis (unweighted UniFrac distance metric) from 16S rRNA gene sequence data shows no difference among normal sleepers (1) and short sleepers (0) and long sleepers (2) in each country site. (**A**) USA, the United States of America, (**B**) RSA, South Africa (**C**) Ghanaian and (**D**) Jamaican populations.
(JPG)

S5 Fig 16S rRNA gene sequence data shows a country-related physiological or environmental influence change in the host is conducive to a less complex microbiota. (**A**) Alpha diversity analysis (Shannon Index); (**B**) beta diversity analysis (weighted UniFrac distance metric) from *** p < 0.001.
(JPG)

S6 Fig Predicted genes involved in butyrate biosynthesis pathways show that genes involved in the lysine pathway are enriched in short sleepers, compared to normal sleepers and long sleepers. K18012, 3,5-diaminohexanoate dehydrogenase; K18013, 3-keto-5-aminohexanoate cleavage enzyme. * p < 0.05.
(JPG)
