# Supplementary figures and images for: Gut microbiota alterations in response to sleep length among African-origin adults
Source: PLoS One. 2021 Sep 8;16(9):e0255323. doi: 10.1371/journal.pone.0255323 (PMC8425534; doi:10.1371/journal.pone.0255323)

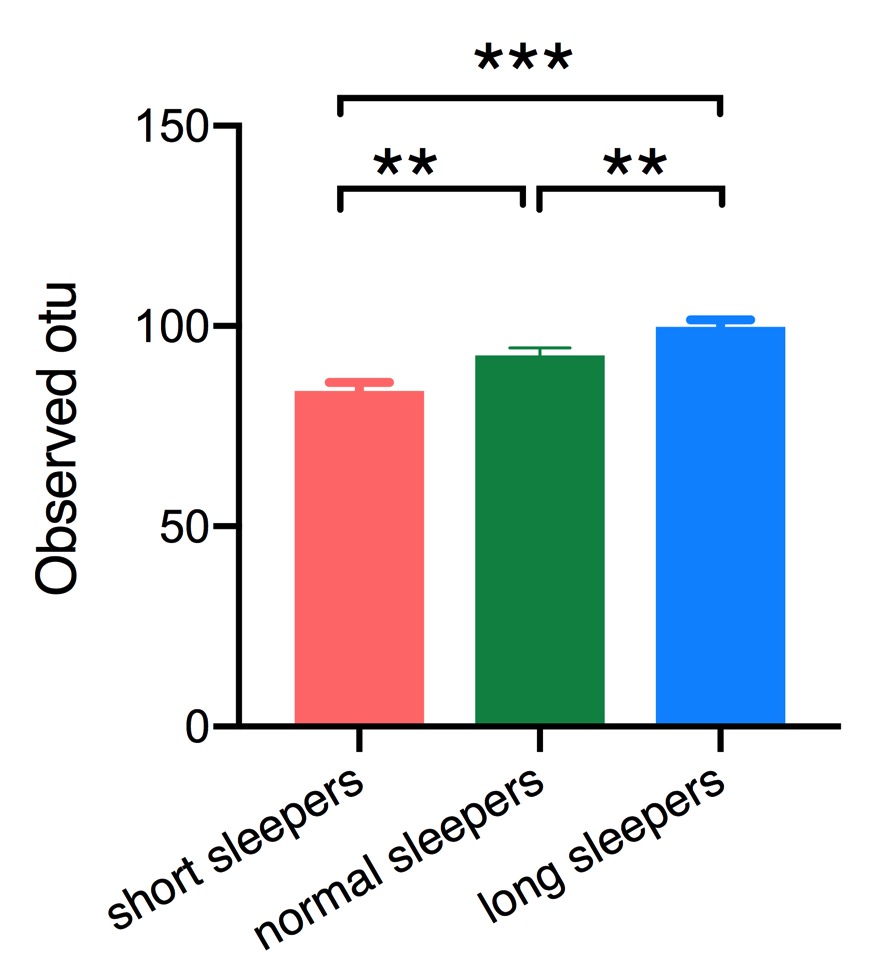

Supplement: S1 Fig — (JPG) [file pone.0255323.s001.jpg]

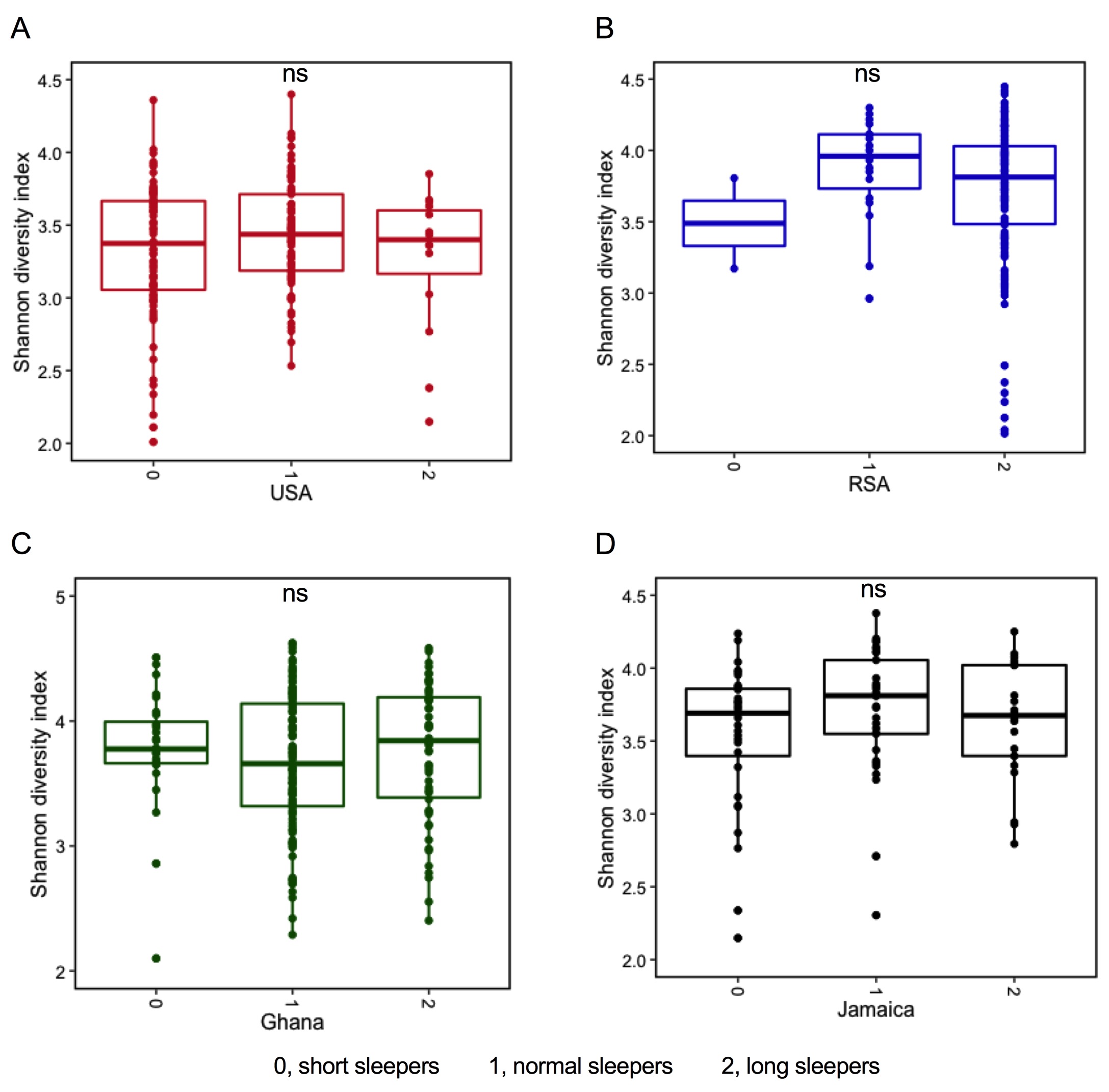

Supplement: S2 Fig — (A) USA, the United States of America, (B) RSA, South Africa (C) Ghanaian and (D) Jamaican populations. (JPG) [file pone.0255323.s002.jpg]

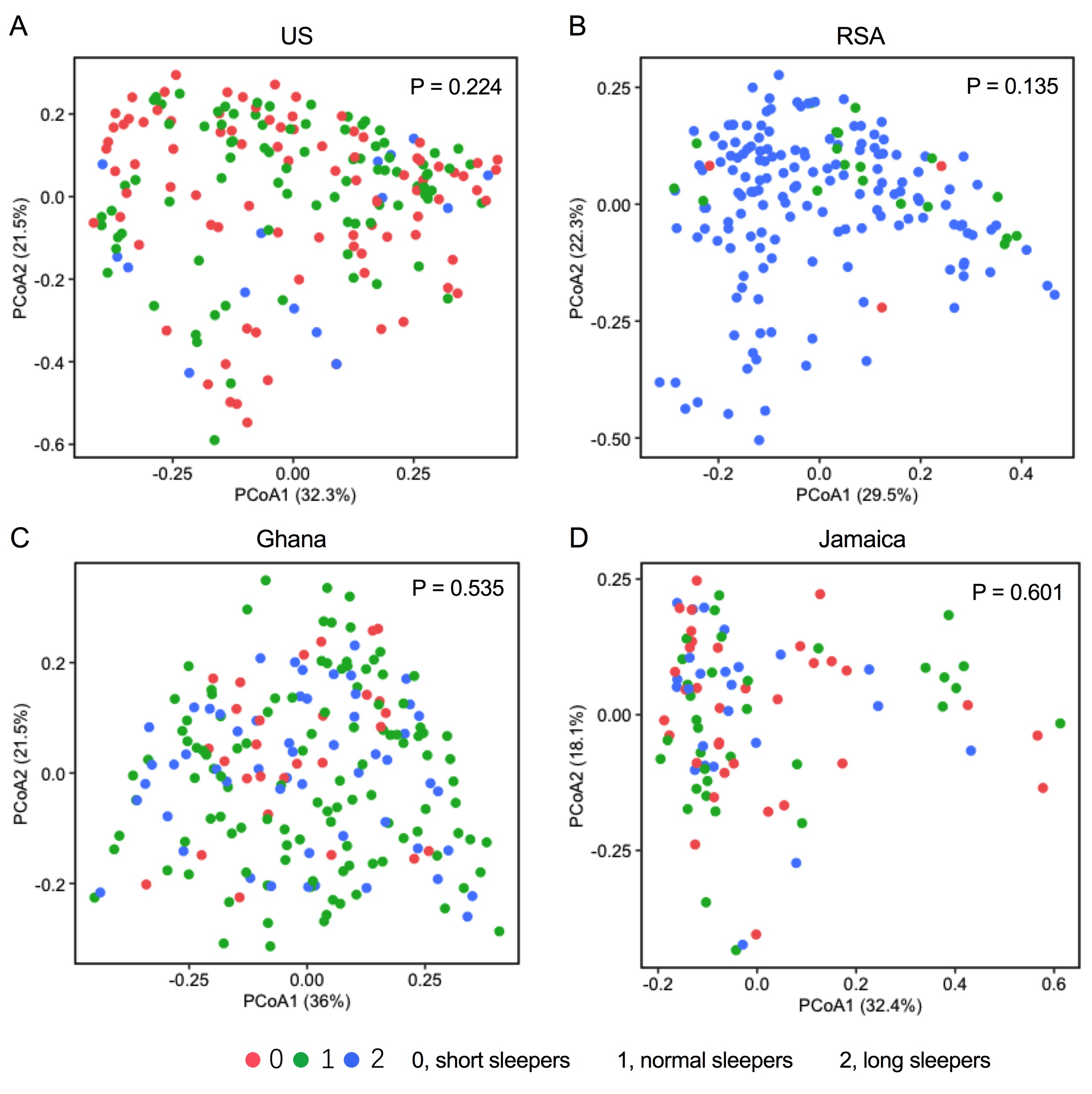

Supplement: S3 Fig — (A) USA, the United States of America, (B) RSA, South Africa (C) Ghanaian and (D) Jamaican populations. (JPG) [file pone.0255323.s003.jpg]

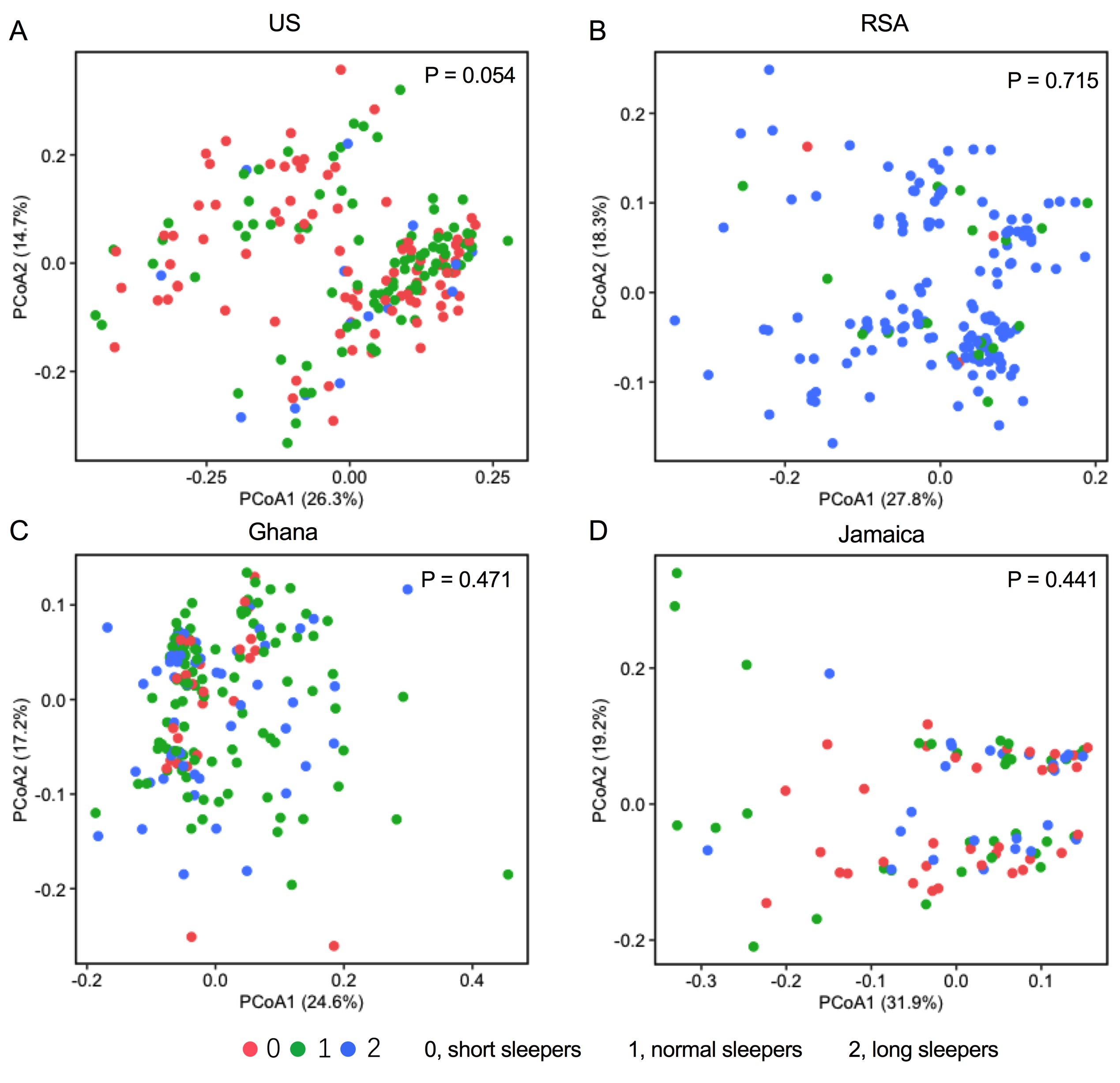

Supplement: S4 Fig — (A) USA, the United States of America, (B) RSA, South Africa (C) Ghanaian and (D) Jamaican populations. (JPG) [file pone.0255323.s004.jpg]

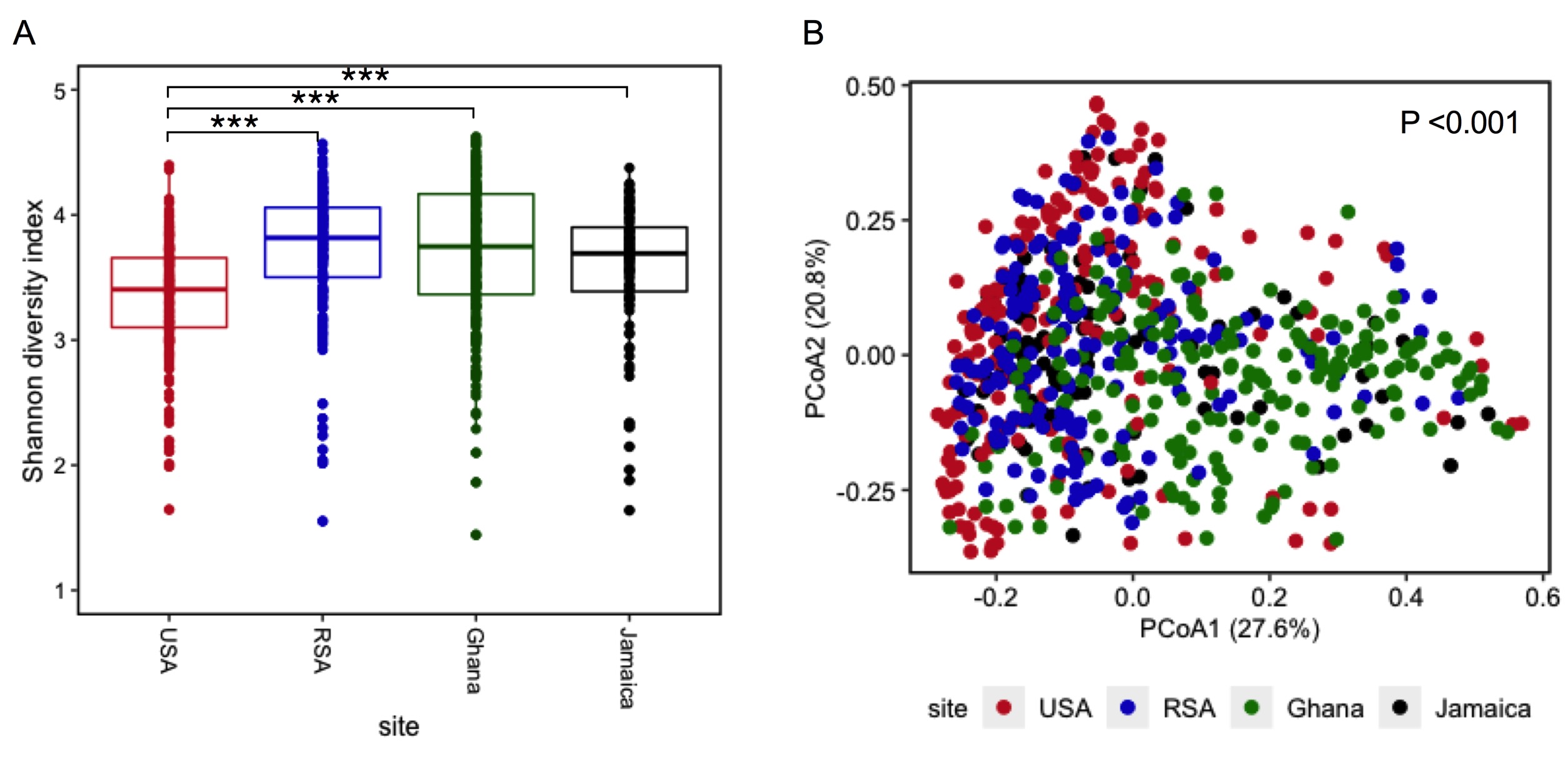

Supplement: S5 Fig — (A) Alpha diversity analysis (Shannon Index); (B) beta diversity analysis (weighted UniFrac distance metric) from *** p < 0.001. (JPG) [file pone.0255323.s005.jpg]

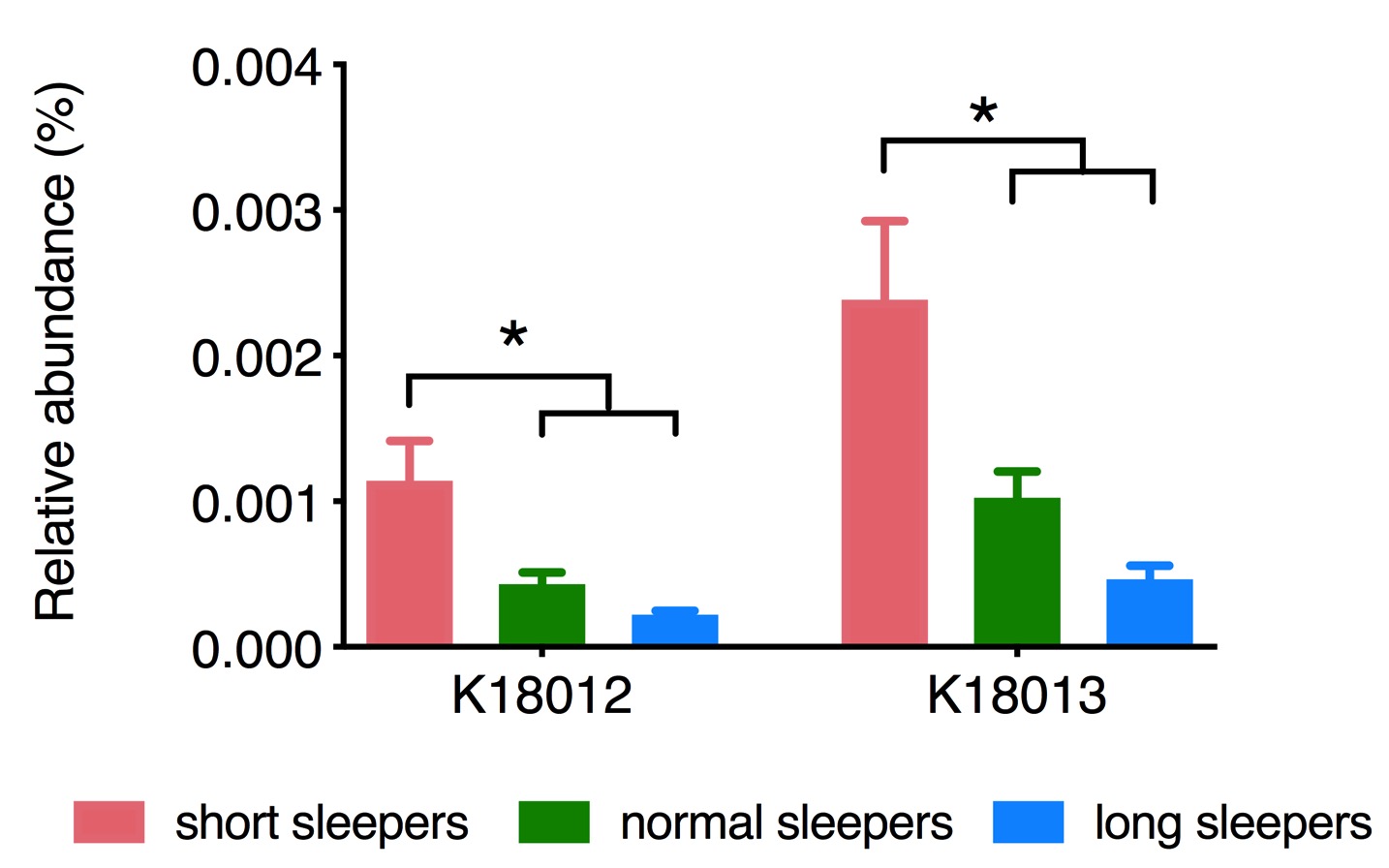

Supplement: S6 Fig — K18012, 3,5-diaminohexanoate dehydrogenase; K18013, 3-keto-5-aminohexanoate cleavage enzyme. * p < 0.05. (JPG) [file pone.0255323.s006.jpg]
